# New growth charts for children and adolescents in Catalonia, Spain: developed from routinely collected data (2013–2019)

**DOI:** 10.1186/s12889-026-27263-x

**Published:** 2026-04-11

**Authors:** Núria Mora, Anna Palomar-Cros, Laura Pérez-Crespo, Leonardo Méndez-Boo, Francisca Ramos, Manuel Quintana, Anna Reñé, Francesc Fina, Ermengol Coma, Talita Duarte-Salles

**Affiliations:** 1 Primary Care Services Information System (SISAP), Institut Català de la Salut (ICS), Gran Via de les Corts Catalanes 587, Barcelona, Spain; 2 Fundació Institut Universitari per a la recerca a l’Atenció Primària de Salut Jordi Gol i Gurina (IDIAPJGol), Gran Via de les Corts Catalanes 587, Barcelona, Spain; 3 Department of Medical Informatics, Erasmus University Medical Center, Dr. Molewaterplein 40, Rotterdam, The Netherlands

**Keywords:** Growth curves, Primary care, Electronic health records, Childhood

## Abstract

**Background:**

Growth charts are essential for detecting early health conditions in children. This study aims to create up-to-date and region-specific growth charts for children and adolescents using large real-world data from primary care electronic health records (EHR) in Catalonia, Spain.

**Methods:**

We used routinely collected EHR of the Institut Català de la Salut, the main primary care provider in Catalonia. We included children aged one month to 14 years with a birthweight greater than 2500 gr, and at least one anthropometric measurement registered (2013–2019). Growth references for head circumference, weight, length/height, and body mass index (BMI) were fitted using GAMLSS models. We built our charts with data recorded in 2013, validated them using data from 2014 to 2019 and compared them to WHO and Spanish growth charts.

**Results:**

With a total of 876,097 records from 155,950 children, we estimated new growth charts. Our curves were higher in the highest percentile, especially for weight and BMI (starting from 4 years old), compared to the WHO standards and for extreme percentiles compared to outdated national charts but were similar to more recent national curves. Validation analyses showed similar results with differences only seen for extreme weight and BMI percentile curves, older ages and later years.

**Conclusions:**

We created new growth charts using a novel approach, accurately representing local growth patterns in the population living in Catalonia. These charts offer primary care professionals a valuable tool for assessing children’s and adolescents’ growth in the region.

**Supplementary Information:**

The online version contains supplementary material available at 10.1186/s12889-026-27263-x.

## Background

Growth is a key indicator of health status in children and adolescents and an important determinant of adult health both at the individual and population level. Deviations from normality might indicate the existence of underlying disease and, thus, its early detection could contribute to reducing morbidity and mortality [1]. Growth charts allow the visualization of anthropometric parameters and are widely used worldwide to assess health status, nutrition, and overall growth of children and adolescents [2–4]. They serve as a screening tool for detecting growth failure and overweight, monitoring the nutritional wellbeing of children, assessing the population wellbeing and evaluating the effectiveness of public health interventions [2].

In 2006, the World Health Organization (WHO) launched international growth charts for all children from birth to 19 years of age [2]. These charts were based on data from children born between 1997 and 2003 in six different countries worldwide: Brazil, United States of America, Ghana, India, Norway and Oman. By 2011, 125 countries worldwide, including Spain, had adopted them [4]. However, controversy has arisen regarding the universal applicability of the WHO charts for monitoring the growth of children and adolescents [5]. Previous studies have found significant differences between the WHO and regional growth charts [6–8]. In fact, a recent meta-analysis covering 55 countries and including 11 million children under five found that 20% of the height measurements exceeded a Z-score of 0.5 SD relative to WHO standards. Children from European cohorts generally had Z-scores above 0.5 SD, while those from Southeast Asian populations typically fell below − 0.5 SD [9]. Therefore, to accurately evaluate child growth parameters, standardized growth charts tailored to regional populations are needed.

The need to establish population-based growth standards has prompted several investigations in Spain [10]. In Bilbao, in 2004, Sobradillo and colleagues published the growth charts from longitudinal and transversal studies, and these have been widely used across Spain since then [11]. These charts were built with data from children born in the 80s. After that, other cross-sectional and longitudinal studies were also conducted in Barcelona, Andalucía and Zaragoza. These studies were integrated into a single study [12, 13], the «Transversal Spanish Study of 2008» which was then updated in 2010 [14] with the addition of data from Madrid. This integration of Spanish data also enabled the publication of the «Longitudinal Spanish Study 1978/2000» [15]. To date, most of these studies included small population sample sizes, were based on data from different healthcare settings, and generally followed heterogenous methodologies for data collection.

In Catalonia, an autonomous region in northeastern Spain, the longitudinal growth charts obtained by Sobradillo and colleagues, from the Faustino Orbegozo Foundation, in 2004, [11] are currently used in routine primary care paediatrician visits and are integrated in the EHR. Nevertheless, these growth charts could be out of date since growth patterns may have changed since the 80s and the Catalan population might be different than the Basque population. This may affect the applicability and validity for monitoring child and adolescent growth of the Catalan population followed in primary care practices. In Catalonia, primary care paediatric services perform a standardized monitoring of child growth as part of a program of preventive activities and health promotion in paediatric age, with high coverage [16]. Thus, periodic measurements of anthropometric parameters such as weight, length/height, body mass index (BMI), and head circumference are recorded routinely in electronic health records (EHR). The routine collection of extensive data in primary care settings offers an opportunity to create new growth charts that accurately represent the local population.

Therefore, the main aim of this study is to generate new child and adolescent growth charts using large real-world data from primary care EHR in Catalonia. The specific objectives are: (i) to generate new references for age- and sex-specific percentiles and z-scores references for weight, length/height, BMI and head circumference; ii) to compare the new growth charts generated in this study with the most commonly used growth charts in Catalonia (Sobradillo 2004 [11], WHO 2006 [2], Carrascosa 2010 [14]).

## Methods

### Study design and data source

We conducted a retrospective population-based cohort study using routinely collected data from the primary care EHR of the Institut Català de la Salut (Catalan Institute of Health; or ICS, for its initials in Catalan). ICS is the main primary care provider in Catalonia and manages around 75% of all primary care practices in the Catalan public health system. ICS gives coverage to approximately 5.8 million people (including around 800,000 children under 15 years old). Its demographic is highly representative of the population of Catalonia in terms of geographic, age and sex distributions [17]. All primary care professionals in the ICS primary care practices use the same EHR system known as ECAP. ECAP is a software used since 2006 that serves as a repository for structured healthcare data including diagnoses, treatments and clinical variables/measurements (e.g.: weight, length/height).

### Study population and study period

In this study we included all children assigned to a primary care practice of the ICS aged one month to 14 years with a birthweight greater than 2500 gr, and at least one weight, length/height, BMI or head circumference measurement recorded between 2013 and 2019.

### Anthropometric measurements and other variables

Body length/height (cm) and weight (kg), BMI (kg/m^2^) as well as head circumference (cm), are routinely measured by nurses in primary care practices following the same protocol [16]. Head circumference is measured from zero to 12/15 months of age while length/height and weight are measured from zero to 14 years of age and BMI from three/four years of age and until 14 (Table 1).

**Table 1:** Ages included in the Catalan program of preventive activities and health promotion in paediatric age and in fitted models

Variable	0–2 years		4–14 years	
	Catalan protocol	Fitted models	Catalan protocol	Fitted models
Weight	7 days, 1, 2, 4, 6, 9, 12, 18 and 24 months	1, 2, 4, 6, 12, 18 and 24 months	36, 48, 72, 96, 120, 144, 168 months	48, 72, 96, 120, 144, 168 months
Length/height	7 days, 1, 2, 4, 6, 9, 12, 18 and 24 months	1, 2, 4, 6, 12, 18 and 24 months	36, 48, 72, 96, 120, 144, 168 months	48, 72, 96, 120, 144, 168 months
Head circumference	1,2,6,9,12,18 and 24 months	1, 2, 4, 6, 12, 18 months	-	-
BMI	-	-	36, 48, 72, 96, 120, 144, 168 months	48, 72, 96, 120, 144, 168 months

Sex assigned at birth, date of birth and nationality (Spanish or non-Spanish) were extracted from primary care data. Age (months) at anthropometric measurements was calculated from the difference between date of each data collection and date of birth. Socioeconomic status in urban areas was assessed using the validated ecological deprivation index based on census data (MEDEA deprivation index) [18]. We categorized this index into four groups, where the first and fourth groups are the least and the most deprived areas, respectively. Rurality was assessed according to whether the primary care practice of the child is from an area with less than 10,000 inhabitants and a population density lower than 150 inhabitants/km^2^, as per regional guidance.

### Growth charts construction and statistical analyses

For the construction of the growth charts, we defined a reproducible methodology for calculating and updating them based on five key steps: data extraction, cleaning process, selection of ages to fit the curves, statistical analysis, and validation.

#### Data extraction

We extracted data from the primary care EHR spanning from January 1st 2013 to 31st December 2019. This data was routinely collected by primary care professionals during their clinical practice. We used data from 2013 to estimate the growth charts and data from 2014 to 2019 for validation. After examining the distribution of anthropometric measurements by year, we found them to be very similar (see Supplementary Fig. 1). As a result, we decided to calculate the growth curves using only the 2013 data.

#### Cleaning process

Individual data underwent a data-cleaning process to detect and remove biological implausible values of height, weight, BMI and head circumference. Errors and implausible values were detected based on the 1977 National Center for Health Statistics and World Health Organization (NCHS/WHO) guideline according to fixed exclusion range, as raw weight-for-age Z-scores lower than − 6.0 or higher than + 5.0, raw height-for-age z-scores lower than − 6.0 or higher than + 6.0, raw head circumference-for-age z-scores lower than − 5.0 or higher than + 5.0, and raw BMI-for-age z-scores lower than − 4.0 or higher than + 5.0 [2].

We excluded children with an excessive number of measurements after 6 months, assuming they likely reflect an underlying medical condition that could affect growth. We defined this threshold as ± 3×IQR (interquartile range) of the distribution of the number of measurements performed between 6 and 12 months and every year until 15 years, as reported in the literature [19, 20]. The defined threshold for each measurement and age is presented in Supplementary Table 1. The number of children excluded because they had an excessive number of measurements is indicated in Supplementary Table 2.

#### Selection of ages to fit the curves

For each specific age (in months) and anthropometric measurement, we assessed the frequency of measurements. We selected the ages with the highest absolute frequency for inclusion. Supplementary Fig. 2 illustrates the frequency distribution of all measurements and highlights the selected ages.

To ensure that the data reflected measurements from healthy children, rather than those with underlying conditions that might affect growth and result in more frequent measurements, we cross-checked our selected ages with those specified in the standardized protocol for preventive activities and health promotion in the Childhood with Health program by the Catalan Health Department [16]. In most cases, the ages we included for our growth charts aligned with the scheduled follow-up visits for healthy children according to this protocol (Table 1).

Given the higher frequency of measurements taken between birth and 2 years of age compared to those taken after 2 years, we decided to estimate growth curves using two separate models: one model for measurements up to 24 months and another for measurements starting from 4 years of age. The specific ages included in each model are listed in Table 1. To construct the global growth curve, we combined the adjusted data points from each model, corresponding to the respective age ranges.

#### Statistical analysis

Growth references for head circumference, weight, length/height, and BMI for age were fitted using generalized additive models for location, scale, and shape (GAMLSS), using R version 4.3.3 and the LMS function from GAMLSS 5.4-22 package [21]. GAMLSS models assume that the response variable follows a parametric distribution y ~ D(μ, 𝛔, ᵞ, τ), where the parameters of the distribution can be modelled, each independently, using non-parametric functions (linear, additive, or nonlinear) [21, 22].

The LMS function works with iterations by adjusting a subset of GAMLSS distributions and compares the goodness of fit using the GAIC (generalized Akaike information criterion to achieve the best model. These distributions include the Box-Cox Cole and Green distribution (BCCG) [23], Box-Cox power exponential (BCPE) [24] and the Box-Cox t (BCT) [25]. The LMS function uses s-splines to smooth the fitting functions of the distribution parameters [21].

The internal fit of each model was checked through visual inspection of worm plots [26].

#### Validation and comparison with other growth charts

Validation of the results was performed through visual inspection using data from 2014 to 2019 to overlay the observed values during these years on the fitted curves. The plotted values included the P3, P50 and P97 percentiles.

We compared the results from the generated charts in 2013 with existing growth charts (Sobradillo 2004 [11], WHO 2006 [2], Carrascosa 2010 [14]). This comparison involved a visual inspection of the P3, P50, and P97 percentiles for each growth chart. Additionally, we calculated the relative differences between our curve and the existing ones for all ages available from the WHO 2006, Sobradillo 2004, and Carrascosa 2010. The relative difference was obtained by subtracting the ECAP value from the value of the curve being compared, dividing by the value of the curve being compared, and then multiplying by 100.

## Results

**Fig. 1:**
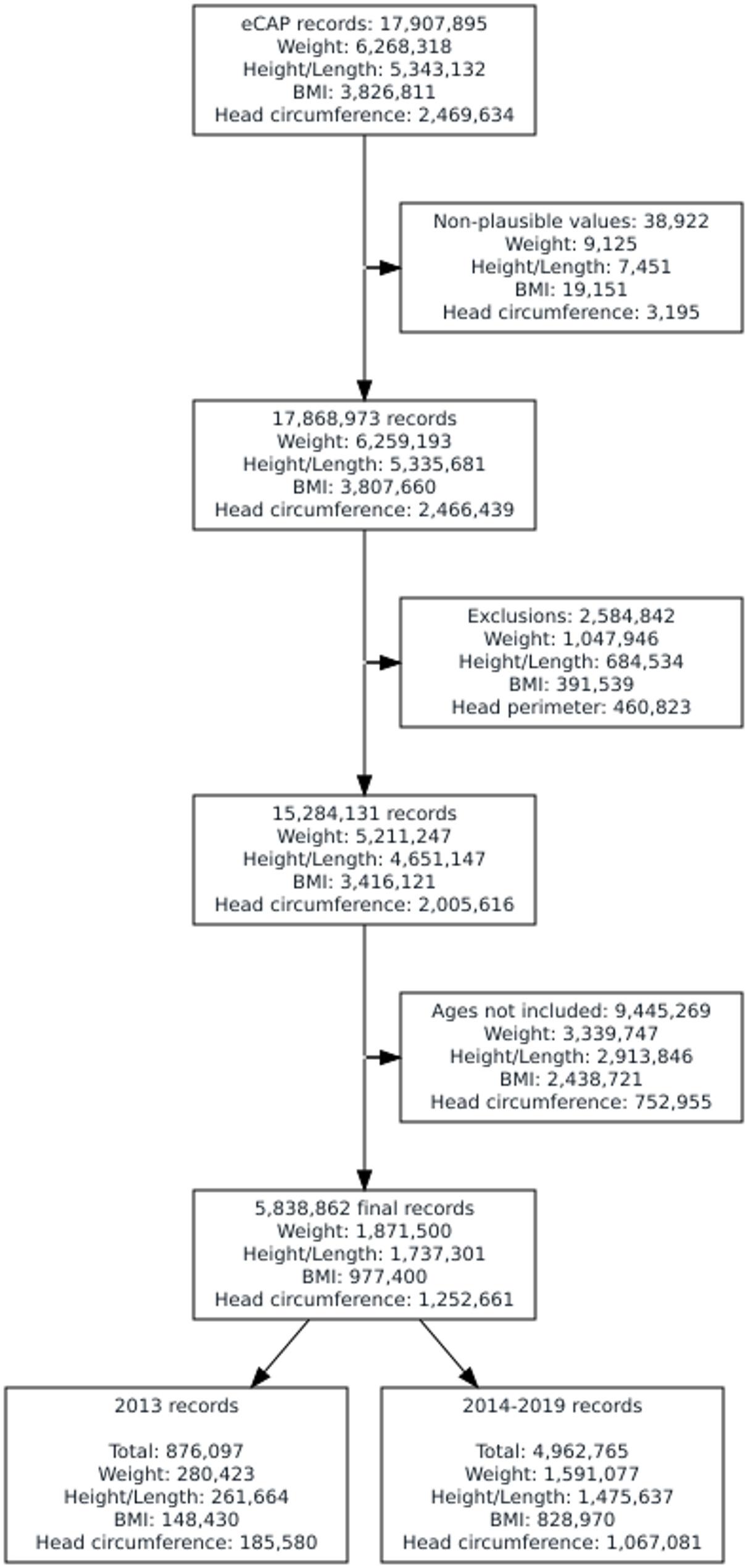
Flow chart of measurements used to construct the growth charts

In our study, we extracted 17,907,895 records (6,268,318 of weight, 5,343,132 of length/height, 3,826,811 of BMI, and 2,469,634 of head circumference) registered in the primary care EHR between the years 2013 and 2019, corresponding to 1,135,602 children under 15 years old (Fig. 1). We removed implausible values (*N* = 38,922 records). Supplementary Fig. 3 illustrates the distribution of included and excluded records for weight, height/length, BMI, and head circumference after this cleaning process. We also excluded records from children with an excessive number of measurements (*N* = 2,584,842 records). Finally, the age selection process to fit the curves (as indicated in Supplementary Fig. 2) excluded 9,445,269 records from 1,034,119 children, which resulted in the inclusion of 1,871,500 weight records, 1,737,301 height/length records, 977,400 BMI records, and 1,252,661 head circumference records from 560,476 children.

A total of 876,097 records from 155,950 children registered in the primary care EHR in 2013 were used to estimate the new growth charts. A similar volume of data and distribution from 2014 to 2019 was used for validation purposes (Table 2, Supplementary Table 3). The percentage of females in 2013 was 48.6%, 27.59% of the children were from rural areas, and 22.4% were from the most deprived areas. These percentages remained stable throughout the years. Conversely, the percentage of the non-Spanish population increased from 6.89% in 2013 to 10.59% in 2019 (Table 2). The baseline characteristics of the included sample (the total for all years) was similar in terms of geographic representation across Catalonia, socioeconomic status, nationality and sex with the initial sample considered (Supplementary Table 4).

**Table 2:** Baseline characteristics of the study population used to estimate and validate the growth charts

Variable​	2013​	2014​	2015​	2016​	2017​	2018​	2019​
N children	155,950​	158,740​	150,746​	148,745​	140,953​	135,277​	129,077​
Socioeonomic status (%)							
Rural​ areas	43,034​ (27.59)​	45,063​ (28.39)​	42,980​ (28.51)​	42,224​ (28.39)	39,610​ (28.10)​	38,373​ (28.37)​	36,669​ (28.41)​
Urban least deprived	20,325​ (13.03)​	19,857​ (12.51)​	18,697​ (12.40)​	18,513​ (12.45)​	17,412​ (12.35)​	16,583​ (12.26)​	15,951​ (12.36)​
Urban 2Q	24,156​ (15.49)​	23,404​ (14.74)​	21,344​ (14.16)​	21,459​ (14.43)​	20,473​ (14.52)​	19,420​ (14.36)​	18,291​ (14.17)​
Urban 3Q	33,485​ (21.47)​	36,548​ (23.02)​	34,002​ (22.56)​	33,491​ (22.52)​	32,529​ (23.08)​	30,860​ (22.81)​	29,669​ (22.99)​
Urban most deprived	34,950​ (22.41)​	33,868​ (21.34)​	33,723​ (22.37)​	33,058​ (22.22)​	30,929​ (21.94)​	30,041​ (22.21)​	28,497​ (22.08)​
Nationality (%)							
Spanish	145,208​ (93.11)​	147,917​ (93.18)​	140,436​ (93.16)​	138,105​ (92.85)	129,687​ (92.01)​	122,879​ (90.84)​	115,409​ (89.41)​
Non-Spanish	10,742​ (6.89)​	10,823 ​ (6.82)​	10,310​ (6.84)​	10,640​ (7.15)​	11,266​ (7.99)​	12,398​ (9.16)​	13,668​ (10.59)​
Moroccan	3,105 (1.99)	3,068 (1.93)	2,891 (1.92%)	2,923 (1.97)	2,993 (2.12)	3,576 (2.64)	4,138 (3.21)
Romanian	2,003 (1.28)	2,083 (1.31)	2,084 ( 1.38)	2,199 (1.48)	2,154 (1.53)	2,097 (1.55)	1,961 (1.52)
Chinese	1,223 (0.78)	1,273 (0.80)	1,121 (0.74)	1,114 (0.75)	1,101 (0.78)	1,148 (0.85)	1,030 (0.80)
Pakistani	421 (0.27)	376 (0.24)	378 (0.25)	395 (0.27)	465 (0.33)	588 (0.44)	706 (0.55)
Italian	241 (0.16)	251 (0.16)	247 (0.16)	219 (0.15)	256 (0.18)	269 (0.20)	300 (0.23)
Bolivian	231 (0.15)	227 (0.14)	177 (0.12)	214 (0.14)	223 (0.16)	208 (0.15)	223 (0.17)
Ecuadorian	222 (0.14)	184 (0.12)	158 (0.11)	144 (0.10)	148 (0.11)	142 (0.11)	136 (0.11)
Senegalese	195 (0.13)	209 (0.13)	186 (0.12)	189 (0.13)	216 (0.15)	259 (0.19)	316 (0.25)
Ukrainian	189 (0.12)	218 (0.14)	256 (0.17)	253 (0.17)	257 (0.18)	250 (0.19)	290 (0.23)
Indian	180 (0.12)	168 (0.11)	190 (0.13)	187 (0.13)	216 (0.15)	257 (0.19)	310 (0.24)
Other	2,732 (1.75%)	2,766 (1.74%)	2,622 (1.74%)	2,803 (1.88%)	3,237 (2.30%)	3,604 (2.66%)	4,258 (3.30%)
Sex (%)							
Male	80,157​ (51.40)​	81,684​ (51.46)​	77,684​ (51.53)​	76,712​ (51.57)​	72,405​ (51.37)​	69,493​ (51.7)​	66,508​ (51.53)​
Female	75,793​ (48.60)​	77,056​ (48.54)​	73,062​ (48.47)​	72,033​ (48.43)​	68,548​ (48.63)​	65,784​ (48.63)​	62,569​ (48.47)​

**Fig. 2:**
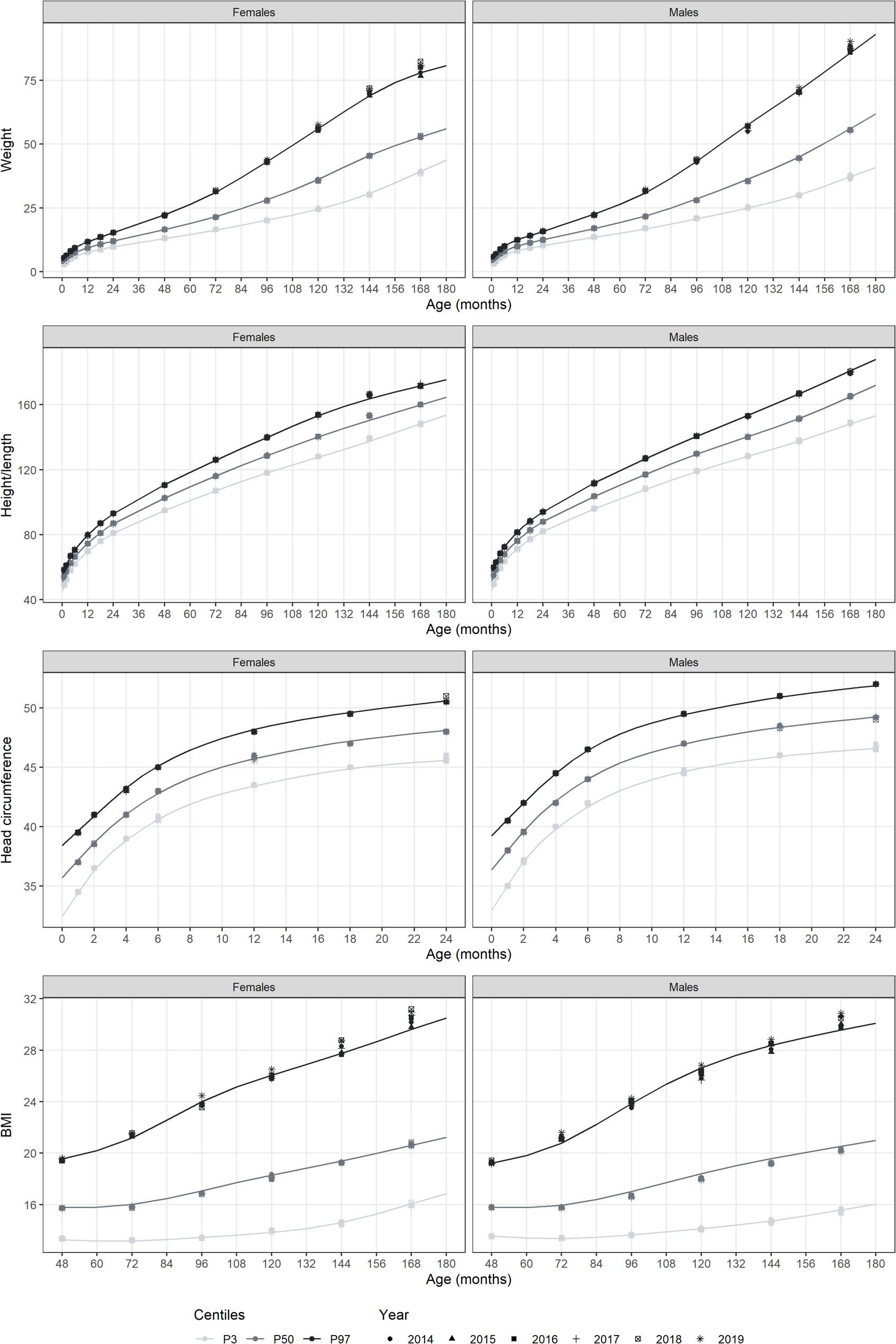
Height, weight, head perimeter and BMI percentile curves for males and females, and validation data from 2014 to 2019. Data are shown for ages one month to 14 years. Weight is measured in kg, height and head perimeter in cm, and BMI in kg/m2

**Fig. 3:**
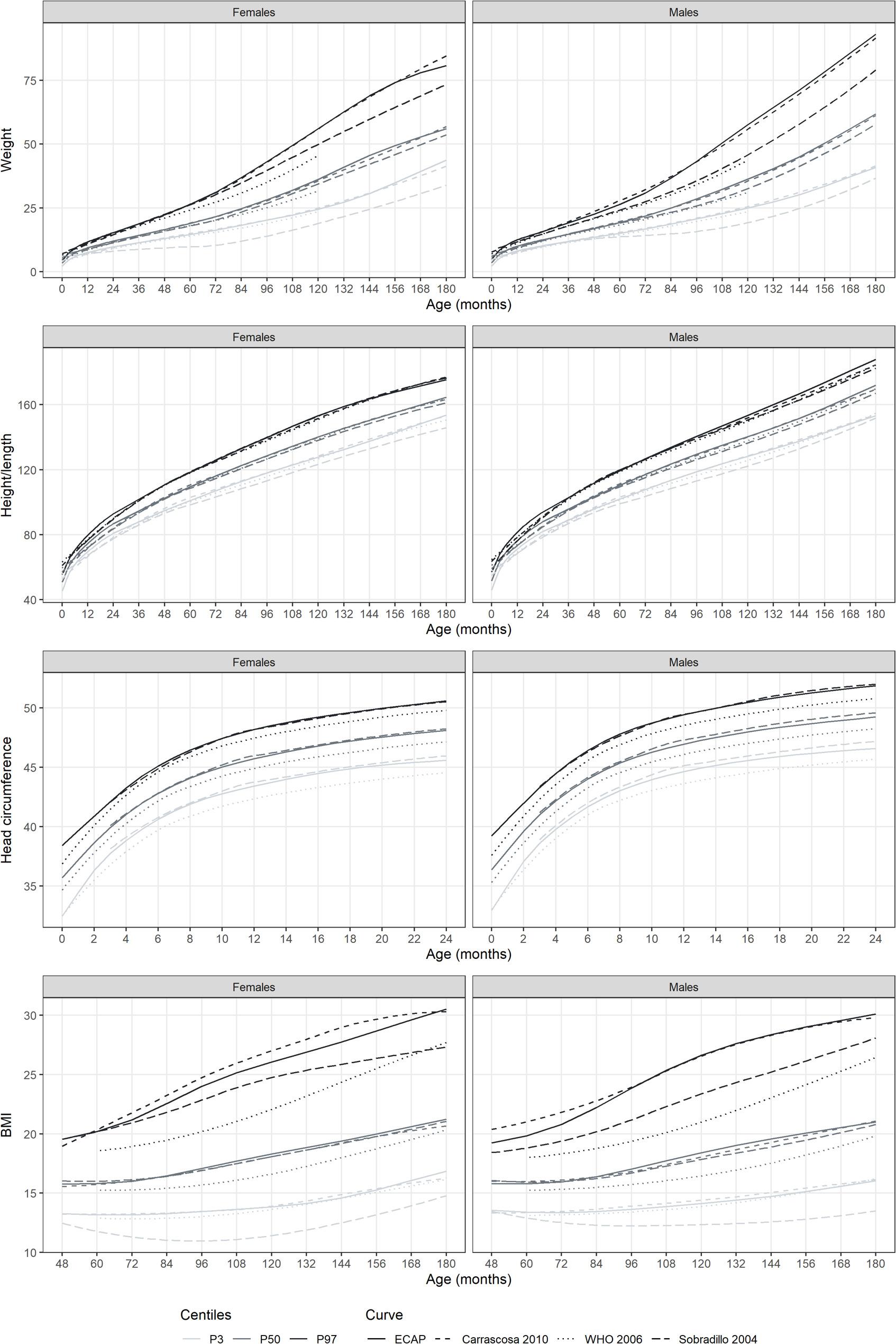
Height, weight, head perimeter and BMI percentile curves for males and females, and percentiles of the existing curves in Spain (Sobradillo 2004, WHO 2006, Carrascosa 2010). Data are shown for ages one month to 14 years. Weight is measured in kg, height and head perimeter in cm, and BMI in kg/m2

Figure 2 and Fig. 3 show the new growth charts for males and females, along with the values from 2014 to 2019, and the percentiles of three of the existing growth charts used in Spain (Sobradillo 2004 [11], WHO 2006 [2], Carrascosa 2011 [14]), respectively. Weight percentile curves for our new charts were above those of the WHO, for both females and males, especially for percentiles P97 and from 48/60 months of age (four/five years old) (Fig. 3). These differences were further explored in Supplementary Table 5. At 5 years of age, the highest percentile (P97) was an 8% hiher for females, and an 11% higher for males, in our charts compared to the WHO charts. At 10, these differences increased to 23% and 31%, respectively. Our growth charts for weight were also above those of Sobradillo 2004 especially in the highest percentile for both males and females and in P3 especially in females. For P97, our charts estimated a weight 11% higher in girls and 27% higher in boys compared to the charts by Sobradillo. Our results were similar to the weight charts estimated by Carrascosa 2010.

Regarding height/length, overall charts were more similar compared with the results on weight. We mainly observed some slight differences with Sobradillo 2004 with our charts showing higher height/length for both females and males (3% and 5% difference, respectively, at 10 years of age Supplementary Table 5). Our results were fairly similar to those of Carrascosa 2010. We compared the modelled percentiles in 2013 with the validation data (2014–2019) and patterns were maintained with very few differences **(**Fig. 2). Differences were only seen for extreme weight percentile curves, older ages and later years:14 years in males, and 11 and 14 years in females.

When looking at the head circumference, we observed noticeable but minor differences with the WHO charts, with our charts showing a larger perimeter in all percentiles for both sexes (Fig. 3). At 12 months of age, the P50 for head circumference estimated with our charts was 2% higher for both sexes compared to the WHO charts Supplementary Table 5). Our results were similar to Spanish charts (only available for Sobradillo 2004), with slightly higher values of p97 up to 6 months, and slightly lower values of p3 afterwards. The same pattern was observed for consecutive years (2014–2019) (Fig. 2).

BMI percentile curves for our charts were above those of the WHO, especially for percentile P97 and to a lesser extent for P50, both for females and males (Fig. 3). At ten years, the BMI P97 estimated in our chart was an 18% higher in females and 27% higher in males, compared to the WHO chart (Supplementary Table 5). The charts by Sobradillo 2004, showed lower BMI curves for both females and males in the extreme percentiles, P97 and P3. Our estimated P97 for BMI at 14 years old for females was an 11% to the WHO charts. The relative difference in males of 9%. Our results were similar to those of Carrascosa 2010, except for BMI in females, which was slightly lower in our charts. Similarly, in the validation of the BMI curves with data from 2014 to 2019, higher values were observed for years after 2013 in the highest percentile (Fig. 2).

The values of the percentiles of the four new growth charts calculated with data from the primary care EHR are shown in the Supplementary Tables 6, and their corresponding charts for male and female can be seen in Supplementary Figs. 4–15. The values for the z-scores are presented in Supplementary Table 7.

Inspection of worm plots suggested good internal fit (Supplementary Figs. 16–27).

## Discussion

### Main findings

This study is among the first to use large EHR data on anthropometric measurements, routinely collected by primary healthcare professionals, to develop children and adolescents’ growth charts. We successfully generated new growth charts for weight, height and BMI from one month to 14 years of age and for head circumference from one month and up to two years of age. The newly obtained growth curves represent local growth patterns of the population living in Catalonia who were visited at primary care.

Overall, the charts generated in this study were above those of the WHO 2006, especially for weight and BMI and for older ages starting from 4 years old [2]. Compared to the charts by Sobradillo 2004 [11], the growth charts currently used in primary care in Catalonia, our charts on weight and BMI were also above in extreme percentiles. Our charts were fairly similar to those by Carrascosa 2010 [14]. The observed differences across charts indicate that the choice of reference curve can significantly impact the values obtained, highlighting its crucial role in growth monitoring.

We compared the modelled percentiles in 2013 with the validation data (2014–2019) and patterns were mostly maintained with differences only seen for the highest percentile of weight and BMI percentile curves in later years and older ages (12–14 years old). These results demonstrate the consistence and quality of the created curves, but also underscore the importance of periodically updating them.

### Comparison with other growth curves

In the primary care practice of the Catalan public health system, primary care professionals use the charts by Sobradillo and colleagues to monitor [11]. These charts might not be representative and applicable to the current population of children and adolescent living in Catalonia, since they were created based on data from children born in the 80s and from the Basque country. Our generated charts, show higher values for weight in extreme percentiles (P97 and P3) and both in males and females compared with the charts by Sobradillo (2004), starting from 4 years old, and increasing the difference in later ages. The standards by Sobradillo also show lower height values especially in the lowest percentile (P3) for females and males and in higher percentiles for males in older ages (from 10 years old onwards). BMI is also lower both in males and females in extreme percentiles and all ages as estimated with the charts by Sobradillo compared to our curves. In Spain, several studies have previously reported similar differences between region-specific charts and the ones by Sobradillo and colleagues [27–29]. Our results also showed some differences in the percentiles for head circumference estimated in our charts compared to the ones by Sobradillo. In our charts, values were higher especially in the highest percentile from 0 to 8 months and lower for the lowest percentile from 8 months onwards.

These observed differences might be potentially explained by disparities in growth across regions. However, the «Transversal Spanish Study of 2008», which included data from Bilbao, Barcelona, Zaragoza, Andalucía, and Madrid, showed that there are currently no significant differences in height across Spanish regions [14]. In line with this, our results were similar to the growth charts by Carrascosa and colleagues [14], with our charts showing slightly lower values for BMI in females across all ages and in males in younger ages (4–8 years old). Another explanation for the observed differences we observed in comparison to the curves of Sobradillo might be explained by the secular trend in weight and height observed in Spain, and other developed countries, in the last century [10]. This would suggest that growth curves that were based on data from children born in the 1980s are not suitable for children born today. Considering the time trends, some authors suggest that it would be appropriate to conduct cross-sectional studies for building growth charts every 10–15 years in reference populations [16]. Finally, the observed differences could be explained by the fact that the curves by Sobradillo were constructed from longitudinal data whereas our references were built from transversal data.

After the WHO published international growth charts [2] in 2006, a debate emerged within the scientific community about which types of growth charts doctors should use to monitor children’s growth. Since then, hundreds of countries around the world adopted them as the reference for monitoring the growth of children and adolescents [4]. However, multiple studies have reported significant differences between the WHO and regional growth charts, questioning its universal applicability [9]. The WHO growth chart included only one European country, Norway, and a cross-sectional study in Spain has shown that there are differences in weight between children living in Spain or Northen European countries [10]. Some authors argue that growth trends and body mass index (BMI) differences are specific to each local population due to differences in genetic, cultural, and socioeconomic factors that influence physical growth and biological maturation [30]. In fact, compared to the WHO growth charts, our charts show higher values for males and females and especially in higher percentiles of the curve (P97), and head circumference in all percentiles in our study population. These differences [31, 32], including in a study in Spain [33]. Overall, the transition to the international growth standards developed by the WHO may have delayed the detection and underestimated the prevalence of health conditions [5].

To our knowledge, only two other studies have used electronic health records data to generate growth charts for weight and height [20] and for head circumference [19]. Before [20], growth charts were usually produced through ad-hoc studies, including a median of 17,000 children. In this study, we were able to include over 850,000 records from more than 150,000 children registered in the primary care EHR in 2013. Our sample size was similar to the ones in the other two French studies which also used EHR data [19, 20].

### Strengths and limitations

We used a reproducible, efficient, and cost-effective process to update the growth charts for children in Catalonia, leveraging real-world data from the entire Catalan population, which included over 850,000 anthropometric measurements from more than 155,000 children—significantly larger than the sample sizes of other studies. Our method efficiently extracted and analysed this vast amount of data, potentially reducing future costs and time for updates. A major strength of this study is the use of extensive data from the ICS, the main primary care provider in Catalonia, known for its high coverage and representativeness, ensuring robust and applicable results for most of the paediatric and adolescent population in the region. Another strength is that the growth charts developed can be integrated into electronic health record systems, such as ECAP, allowing for the automatic generation and application of growth curves within clinical practice. Finally, using advanced statistical models (GAMLSS) allowed for precise and flexible adaptation of the growth curves to the data.

However, it is important to acknowledge some limitations of the study. The accuracy and reliability of measurements may be affected by variability in the instruments used by primary care professionals, although a common protocol is followed, and scales are routinely calibrated. Therefore, measurement error is likely to be non-differential. Another limitation of our study is that the selected ages for growth chart development were based on the frequency of measurements, which could potentially include data from children with underlying conditions affecting growth. We addressed this by cross-referencing our selected ages with the standardized protocol for preventive activities and health promotion in the Childhood with Health program, ensuring that most of the ages aligned with scheduled follow-up visits for healthy children. Although the ages selected in standardized protocols cover the key stages of growth and allow for the development of practical and reliable growth charts, they do not capture every point of adolescence due to logistical and resource constraints. Measurements at 12 and 14 years approximate the average onset and mid-phase of puberty, when the pubertal growth spurt occurs. Nevertheless, by constructing continuous growth charts, it is possible to extrapolate intermediate values, while keeping in mind that individual variation in pubertal timing should be considered when interpreting the results. Additionally, we excluded children with an unusually high number of anthropometric measurements, using this as a proxy to identify and exclude those with potential health conditions.

The lack of information on variables such as gestational age, breastfeeding, and birth weight for all participants might also limit the interpretation of the results. Nevertheless, the broad coverage and large amount of included data provide a solid basis for the validity of the new growth charts. The demographic change in Catalonia during the study period, with the proportion of children of foreign origin increasing from 6% to nearly 11%, may have introduced additional variability in growth patterns due to differences in genetic and environmental factors. However, this heterogeneity also increases the representativeness of the charts for the current paediatric population in Catalonia. Finally, weight has been increasing over the years due to a rising global burden of overweight and obesity. A potential risk with new growth charts is that they might normalize this excess weight. While the new charts reflect the current growth patterns of the Catalonian population, further studies are needed to establish obesity thresholds based on these charts.

### Implications and future research

The new growth charts developed in this study provide a valuable tool for primary care health professionals in Catalonia, enabling them to more accurately assess the growth of children and adolescents in the region. Implementing these charts could improve the early detection of growth disorders and contribute to the promotion of child health.

Future research should focus on establishing new cut-off points for specific conditions (such as obesity) and determining the optimal frequency for updates. The ability to update reference curves without relying on longitudinal studies, which require years of follow-up, makes this task more feasible and crucial than it was in the past.

## Conclusions

Monitoring weight, height, BMI and head circumference is crucial for the early detection of health conditions that may impact growth in early childhood. Current international and national charts could be out of date since growth patterns show a positive secular trend and may not be applicable to regional specificities. Our study successfully generated new growth charts specific to children and adolescents in Catalonia using available massive routine datasets from medical records. The generated charts show significant differences with international charts, questioning their universal applicability, and also differences with outdated national growth charts currently in use in the region. However, we found no major differences when comparing them with more recent national charts. This underscores the importance of using customized growth charts that are regularly updated.

This study is one of the first to successfully implement an innovative approach using primary care EHR data to generate growth charts, paving the way for optimized and automated growth monitoring in future research.

## Supplementary Information

Supplementary Material 1.

Supplementary Material 2.

Supplementary Material 3.

Supplementary Material 4.

## Data Availability

Data from this study will be made accessible upon reasonable request.

## Abbreviations

BMI: Body mass index
BCPE: Box-Cox power exponential
EHR: Electronic health records
GAIC: Generalized Akaike information criterion
ICS: Catalan Institute of Health
IQR: Interquartile range
WHO: World Health Organization
