# Supplementary material for: New growth charts for children and adolescents in Catalonia, Spain: developed from routinely collected data (2013–2019)
Source: BMC Public Health. 2026 Apr 11;26:1650. doi: 10.1186/s12889-026-27263-x (PMC13195899; doi:10.1186/s12889-026-27263-x)

[Supplementary table 1. Threshold for defining an excessive number of measurements by type of anthropometric measurement and age 3](#_Toc564736433)

[Supplementary Table 2. Excluded records and individuals during selection process following inclusion and exclusion criteria 4](#_Toc1882134807)

[Supplementary Figure 1. Density plot of the annual number of measurements used to estimate and validate the growth charts from 2013 to 2019 6](#_Toc1725444231)

[Supplementary Figure 2. Frequency of measures by age (in months) 6](#_Toc1752983108)

[Supplementary Figure 3. Included (in black) and excluded (in grey) records of weight, height/length, BMI, and head circumference after the cleaning process. 7](#_Toc1570552574)

[Supplementary Table 3. Annual number of measurements and children used to estimate and validate the growth charts stratified by age groups (0 to 2 years and >2 years), from 2013 to 2019 8](#_Toc1582005020)

[Supplementary Table 4. Baseline characteristics of total included sample (2013-2019) versus initial sample 12](#_Toc1521271270)

[Supplementary Figure 4. Male: Length / height 0-15 years 13](#_Toc1785178495)

[Supplementary Figure 5. Male: Length 0-24 months 13](#_Toc1845966356)

[Supplementary Figure 6. Male: Weight 0-15 years 14](#_Toc1934972819)

[Supplementary Figure 7. Male: Weight 0-24 months  14](#_Toc2058725740)

[Supplementary Figure 8. Male: Body mass index 4-15 years 15](#_Toc1617323878)

[Supplementary Figure 9. Male: Head circumference 0-24 months 15](#_Toc254609411)

[Supplementary Figure 10. Female: Length / height 0-15 years 16](#_Toc1683600310)

[Supplementary Figure 11. Female: Length 0-24 months 16](#_Toc1411915012)

[Supplementary Figure 12. Female: Weight 0-15 years  17](#_Toc592949165)

[Supplementary Figure 13. Female: Weight 0-24 months 17](#_Toc316572332)

[Supplementary Figure 14. Female: Body mass index 4-15 years 18](#_Toc2126513626)

[Supplementary Figure 15. Female: Head circumference 0-24 months 18](#_Toc1670211989)

[Supplementary Figure 16. Worm plot: Weight females 0-24 months 19](#_Toc297832498)

[Supplementary Figure 17. Worm plot: Length females 0-24 months 19](#_Toc1036806289)

[Supplementary Figure 18. Worm plot: Head circumference females 0-24 months 20](#_Toc45734335)

[Supplementary Figure 19. Worm plot: Weight males 0-24 months 20](#_Toc1492253275)

[Supplementary Figure 20. Worm plot: Length males 0-24 months 21](#_Toc1080203453)

[Supplementary Figure 21. Worm plot: Head circumference males 0-24 months 21](#_Toc352132430)

[Supplementary Figure 22. Worm plot: Weight females 4-14 years 22](#_Toc712564478)

[Supplementary Figure 23. Worm plot: Height females 4-14 years 22](#_Toc1981508707)

[Supplementary Figure 24. Worm plot: BMI females 4-14 years 23](#_Toc1000733748)

[Supplementary Figure 25. Worm plot: Weight males 4-14 years 23](#_Toc1348529540)

[Supplementary Figure 26. Worm plot: Height males 4-14 years 24](#_Toc204465397)

[Supplementary Figure 27. Worm plot: BMI males 4-14 years 24](#_Toc1027866276)

# Supplementary table 1. Threshold for defining an excessive number of measurements by type of anthropometric measurement and age

| Periods | BMI | Head circumference | Weight | Height/length |
| --- | --- | --- | --- | --- |
| Between 6 and 12 months | 5 | 5 | 5 | 5 |
| Between 13 and 24 months | 5 | 8 | 6 | 6 |
| Between 2 and 4 years | 8 |  | 5 | 8 |
| Between 5 and 8 years | 8 |  | 8 | 8 |
| Between 9 and 12 years | 11 |  | 8 | 11 |
| Between 13 and 14 years | 11 |  | 11 | 8 |

# **Supplementary Table 2**. Excluded records and individuals during selection process following inclusion and exclusion criteria

| Measurement | Year | A | B | C | D | E | F | G | H | I | J | K | L | M | N |
| --- | --- | --- | --- | --- | --- | --- | --- | --- | --- | --- | --- | --- | --- | --- | --- |
| BMI | 2013 | 537090 | 378627 | 534019 | 377171 | 485295 | 359767 | 22786 | 16198 | 369 | 152 | 25569 | 8747 | 148430 | 117891 |
| BMI | 2014 | 569128 | 400140 | 566030 | 398743 | 506026 | 377767 | 24501 | 17648 | 1361 | 474 | 34142 | 11369 | 152732 | 120910 |
| BMI | 2015 | 570390 | 403069 | 567405 | 401737 | 506226 | 379353 | 24460 | 17667 | 1911 | 818 | 34808 | 12641 | 146598 | 114517 |
| BMI | 2016 | 565437 | 403139 | 562590 | 401881 | 501186 | 378211 | 25096 | 18139 | 2300 | 1072 | 34008 | 13202 | 144327 | 113003 |
| BMI | 2017 | 545028 | 392659 | 542536 | 391515 | 484031 | 367625 | 23384 | 17183 | 2704 | 1457 | 32417 | 13293 | 135411 | 105931 |
| BMI | 2018 | 527710 | 385071 | 525378 | 383988 | 471443 | 360536 | 22378 | 16349 | 3098 | 1715 | 28459 | 12712 | 128502 | 101583 |
| BMI | 2019 | 512028 | 380442 | 509702 | 379342 | 461914 | 356251 | 21475 | 15959 | 3507 | 2044 | 22806 | 11447 | 121400 | 96463 |
| HC | 2013 | 366414 | 110291 | 365907 | 110237 | 300171 | 102080 | 47031 | 33958 | 1140 | 299 | 17565 | 4642 | 185580 | 88984 |
| HC | 2014 | 378587 | 111854 | 378058 | 111789 | 303986 | 101841 | 51550 | 36894 | 3036 | 739 | 19486 | 5498 | 189445 | 89018 |
| HC | 2015 | 368575 | 108247 | 368111 | 108183 | 296175 | 98488 | 49744 | 35379 | 3493 | 996 | 18699 | 5263 | 184480 | 85905 |
| HC | 2016 | 363613 | 107317 | 363129 | 107252 | 292833 | 98181 | 50768 | 35188 | 3782 | 1092 | 15746 | 4549 | 184410 | 85703 |
| HC | 2017 | 348182 | 104755 | 347735 | 104696 | 281927 | 96344 | 49144 | 34204 | 4081 | 1218 | 12583 | 3761 | 177350 | 83681 |
| HC | 2018 | 332907 | 100432 | 332525 | 100376 | 271681 | 93287 | 46671 | 32028 | 4337 | 1321 | 9836 | 2813 | 169439 | 79684 |
| HC | 2019 | 311356 | 93000 | 310974 | 92943 | 258843 | 87591 | 42533 | 29272 | 4322 | 1285 | 5276 | 1717 | 161957 | 75024 |
| Weight | 2013 | 881383 | 432041 | 880202 | 431572 | 740717 | 411582 | 91049 | 43152 | 1620 | 313 | 46816 | 10160 | 280423 | 155710 |
| Weight | 2014 | 932064 | 455111 | 930743 | 454605 | 768823 | 430743 | 99852 | 46061 | 4093 | 750 | 57975 | 13041 | 288729 | 158519 |
| Weight | 2015 | 924941 | 457614 | 923684 | 457116 | 763986 | 431854 | 96988 | 44361 | 4878 | 1099 | 57832 | 14315 | 277151 | 150516 |
| Weight | 2016 | 921615 | 459097 | 920242 | 458547 | 760538 | 432054 | 98904 | 44511 | 5650 | 1427 | 55150 | 14819 | 275158 | 148560 |
| Weight | 2017 | 890748 | 449264 | 889409 | 448713 | 736310 | 422097 | 95792 | 43010 | 6401 | 1888 | 50906 | 14820 | 260837 | 140751 |
| Weight | 2018 | 867528 | 442334 | 866277 | 441784 | 721531 | 415733 | 93044 | 40976 | 7011 | 2152 | 44691 | 14087 | 248786 | 135110 |
| Weight | 2019 | 850039 | 439175 | 848636 | 438577 | 719342 | 413213 | 88208 | 39251 | 7628 | 2501 | 33458 | 12617 | 240416 | 128885 |
| Height/length | 2013 | 765710 | 415817 | 764530 | 415435 | 672201 | 397205 | 56158 | 39617 | 1223 | 301 | 34948 | 9898 | 261664 | 154539 |
| Height/length | 2014 | 801271 | 435062 | 800132 | 434677 | 692999 | 413013 | 60459 | 42351 | 3203 | 744 | 43471 | 12549 | 268053 | 157189 |
| Height/length | 2015 | 794351 | 435613 | 793213 | 435193 | 687234 | 412526 | 58462 | 40616 | 3900 | 1087 | 43617 | 13673 | 257713 | 149142 |
| Height/length | 2016 | 786362 | 434800 | 785173 | 434388 | 680064 | 410794 | 59505 | 40620 | 4458 | 1395 | 41146 | 14058 | 255314 | 146896 |
| Height/length | 2017 | 757950 | 424340 | 756934 | 423975 | 656372 | 400179 | 57273 | 39413 | 5175 | 1840 | 38114 | 13981 | 241752 | 139156 |
| Height/legnth | 2018 | 731259 | 414350 | 730392 | 414005 | 637463 | 391472 | 54051 | 36857 | 5646 | 2082 | 33232 | 13216 | 230923 | 133369 |
| Height/length | 2019 | 706229 | 406640 | 705307 | 406283 | 624814 | 385337 | 49724 | 34193 | 6060 | 2418 | 24709 | 11638 | 221882 | 127026 |
| A: Number of registries at the beginning of the selection process; B: Number of children at the beginning of the selection process; C: Number of registries without implausible values; D: Number of children without implausible values; E: Number of registries with inclusion criteria (IC); F: Number of children with IC; G: Number of registries with age outside of range; H: Number of children with age outside of range; I: Number of registries with low birth weight; J: Number of children with low birth weight; K: Number of registries with an excessive number of measurements; L: Number of children with an excessive number of measurements; M: Number of registries included in the final sample; N: Number of children included in the final sample. BMI: Body-mass index; HC: Head circumference. | | | | | | | | | | | | | | | |

# Supplementary Figure 1. Density plot of the annual number of measurements used to estimate and validate the growth charts from 2013 to 2019


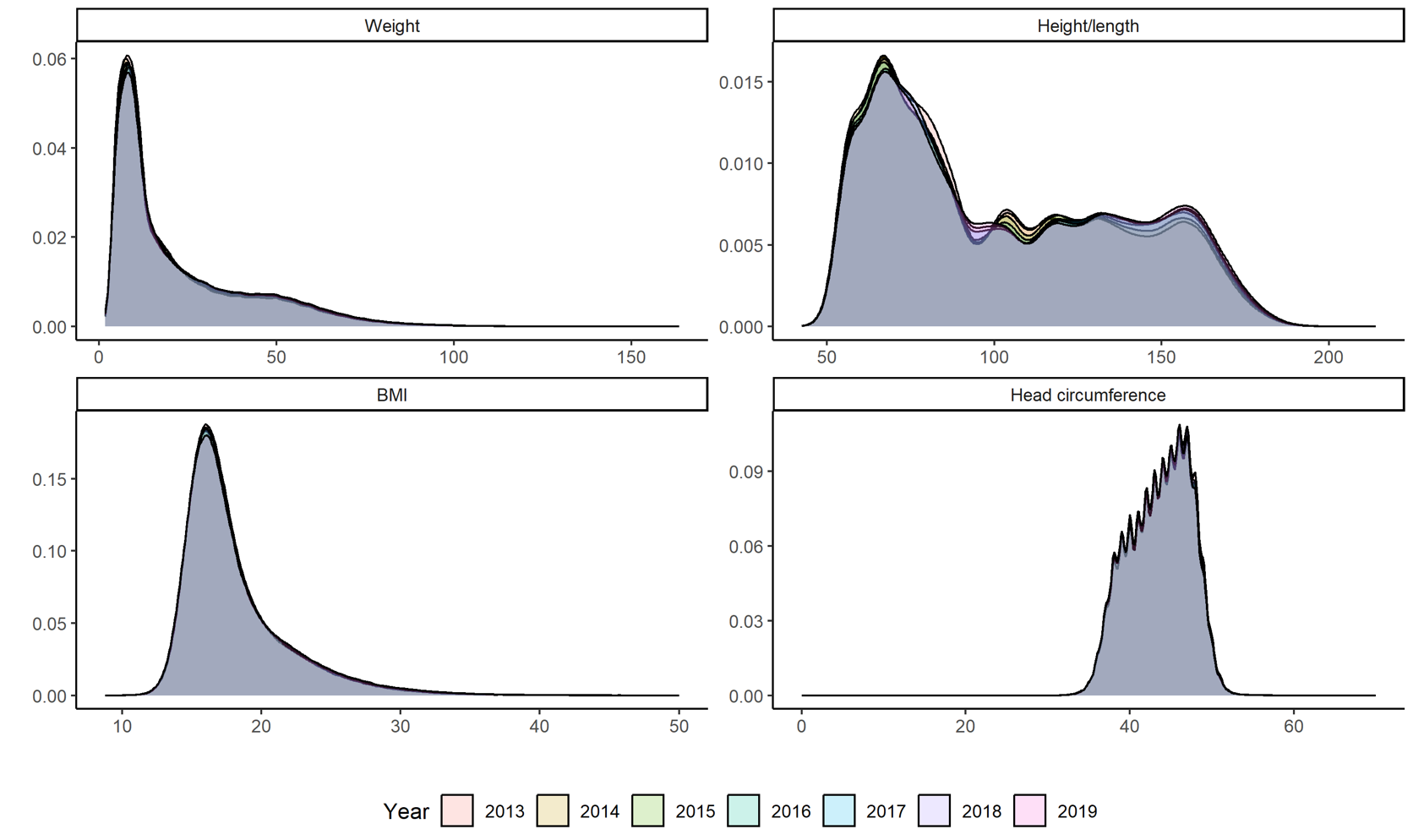
Weight was measured in kg, height/length in cm, BMI in kg/m^2^ and head circumference in cm.

# Supplementary Figure 2. Frequency of measures by age (in months)


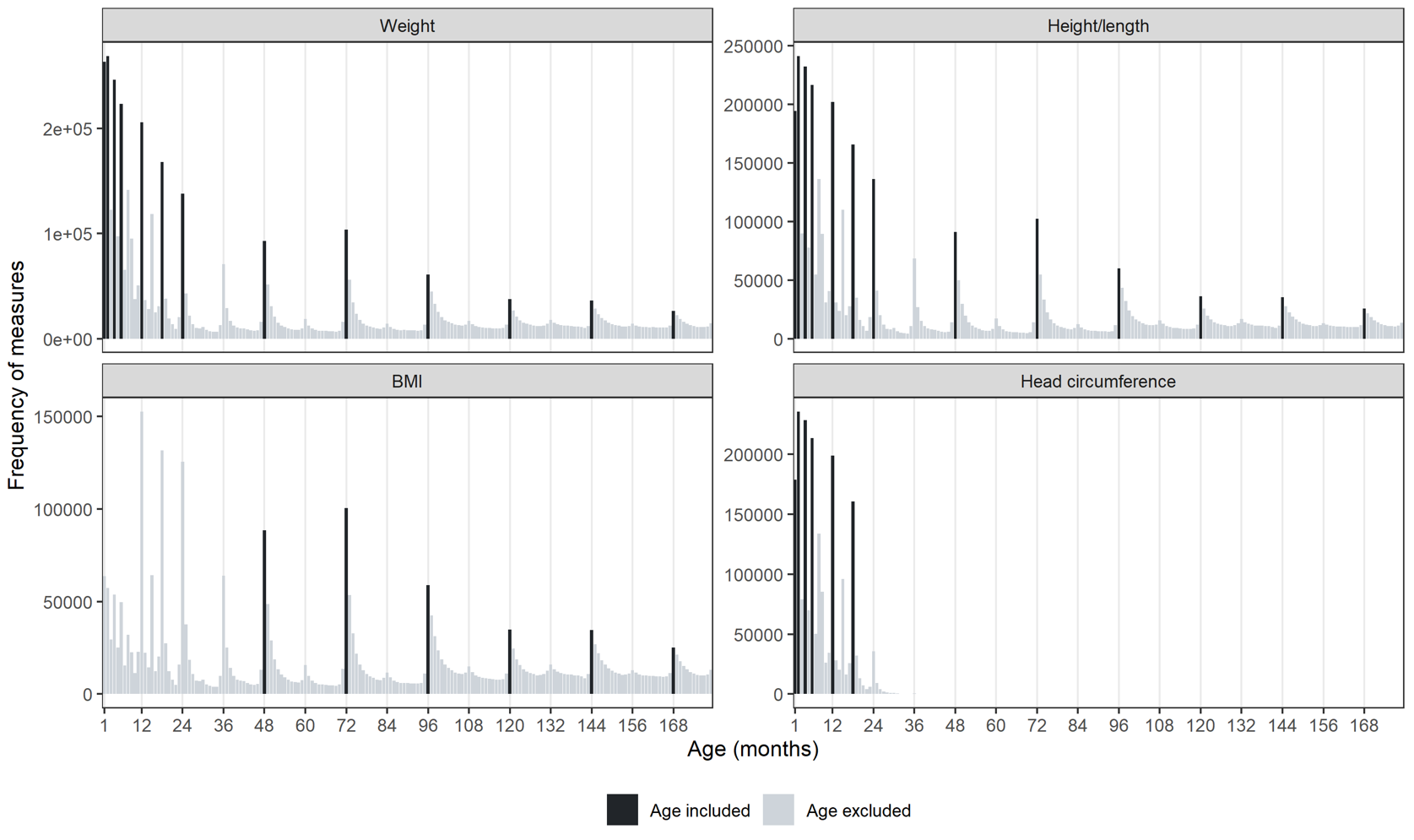


# Supplementary Figure 3. Included (in black) and excluded (in grey) records of weight, height/length, BMI, and head circumference after the cleaning process.


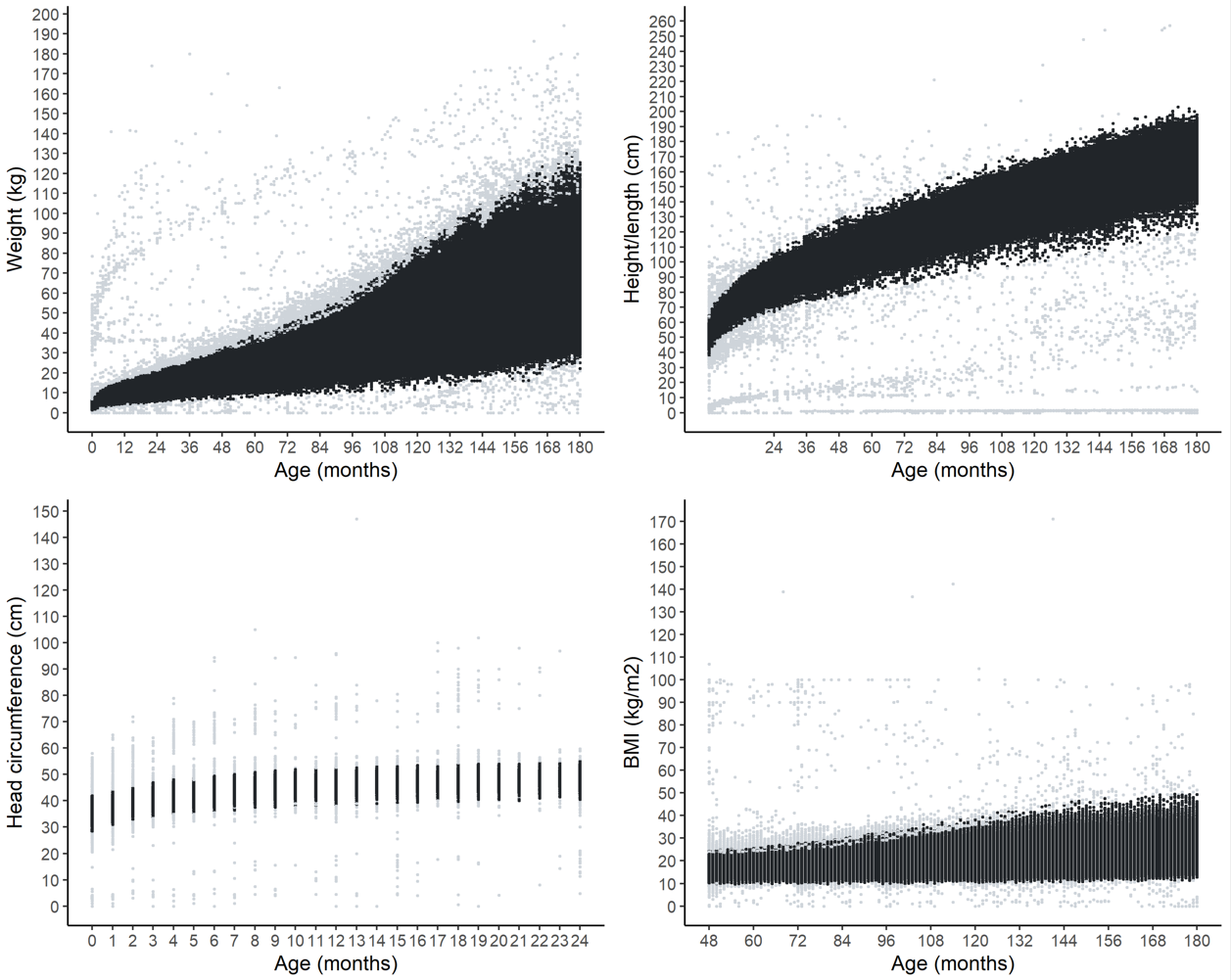


# Supplementary Table 3. Annual number of measurements and children used to estimate and validate the growth charts stratified by age groups (0 to 2 years and >2 years), from 2013 to 2019

| Year | Variable | Sex | Age group | N measurements | N children |
| --- | --- | --- | --- | --- | --- |
| 2013 | Weight | Male | > 2 years | 69926 | 69112 |
| 2013 | Height/length | Male | > 2 years | 69031 | 68629 |
| 2013 | BMI | Male | > 2 years | 60052 | 59670 |
| 2013 | HC | Male | > 2 years | 31935 | 31797 |
| 2013 | Weight | Female | > 2 years | 65774 | 65016 |
| 2013 | Height/length | Female | > 2 years | 64893 | 64518 |
| 2013 | BMI | Female | > 2 years | 56482 | 56107 |
| 2013 | HC | Female | > 2 years | 30138 | 29985 |
| 2013 | Weight | Female | 0-2 years | 71370 | 61024 |
| 2013 | Height/length | Female | 0-2 years | 62594 | 58617 |
| 2013 | BMI | Female | 0-2 years | 15562 | 14694 |
| 2013 | HC | Female | 0-2 years | 60459 | 57402 |
| 2013 | Weight | Male | 0-2 years | 73353 | 63646 |
| 2013 | Height/length | Male | 0-2 years | 65146 | 61304 |
| 2013 | HC | Male | 0-2 years | 63048 | 60047 |
| 2013 | BMI | Male | 0-2 years | 16334 | 15421 |
| 2014 | Weight | Male | > 2 years | 69647 | 68822 |
| 2014 | Height/length | Male | > 2 years | 68670 | 68302 |
| 2014 | BMI | Male | > 2 years | 60560 | 60195 |
| 2014 | Weight | Female | > 2 years | 66015 | 65262 |
| 2014 | Height/length | Female | > 2 years | 65087 | 64729 |
| 2014 | BMI | Female | > 2 years | 57462 | 57093 |
| 2014 | Weight | Male | 0-2 years | 78087 | 67248 |
| 2014 | Height/length | Male | 0-2 years | 68964 | 64658 |
| 2014 | HC | Male | 0-2 years | 66733 | 63304 |
| 2014 | HC | Female | > 2 years | 29106 | 28985 |
| 2014 | Weight | Female | 0-2 years | 74980 | 63606 |
| 2014 | Height/length | Female | 0-2 years | 65332 | 61083 |
| 2014 | HC | Female | 0-2 years | 63076 | 59759 |
| 2014 | BMI | Female | 0-2 years | 16745 | 15661 |
| 2014 | HC | Male | > 2 years | 30530 | 30409 |
| 2014 | BMI | Male | 0-2 years | 17965 | 16892 |
| 2015 | Weight | Female | > 2 years | 62696 | 61941 |
| 2015 | Height/length | Female | > 2 years | 61746 | 61385 |
| 2015 | BMI | Female | > 2 years | 54324 | 53985 |
| 2015 | HC | Female | > 2 years | 28300 | 28171 |
| 2015 | Weight | Male | 0-2 years | 75884 | 65511 |
| 2015 | Height/length | Male | 0-2 years | 67348 | 63171 |
| 2015 | HC | Male | 0-2 years | 65082 | 61823 |
| 2015 | Weight | Male | > 2 years | 66398 | 65593 |
| 2015 | Height/length | Male | > 2 years | 65407 | 64990 |
| 2015 | BMI | Male | 0-2 years | 17902 | 16885 |
| 2015 | BMI | Male | > 2 years | 57501 | 57109 |
| 2015 | HC | Male | > 2 years | 29978 | 29828 |
| 2015 | Weight | Female | 0-2 years | 72173 | 61471 |
| 2015 | Height/length | Female | 0-2 years | 63212 | 59065 |
| 2015 | HC | Female | 0-2 years | 61120 | 57889 |
| 2015 | BMI | Female | 0-2 years | 16871 | 15797 |
| 2016 | Weight | Male | 0-2 years | 76337 | 66002 |
| 2016 | Height/length | Male | 0-2 years | 67538 | 63592 |
| 2016 | HC | Male | 0-2 years | 65419 | 62324 |
| 2016 | Weight | Female | > 2 years | 61258 | 60492 |
| 2016 | Height/length | Female | > 2 years | 60171 | 59799 |
| 2016 | BMI | Female | > 2 years | 53241 | 52906 |
| 2016 | Weight | Male | > 2 years | 65194 | 64378 |
| 2016 | Height/length | Male | > 2 years | 64045 | 63644 |
| 2016 | BMI | Male | > 2 years | 56717 | 56362 |
| 2016 | HC | Male | > 2 years | 29721 | 29604 |
| 2016 | BMI | Male | 0-2 years | 17762 | 16801 |
| 2016 | Weight | Female | 0-2 years | 72369 | 62054 |
| 2016 | Height/length | Female | 0-2 years | 63560 | 59611 |
| 2016 | HC | Female | 0-2 years | 61512 | 58412 |
| 2016 | HC | Female | > 2 years | 27758 | 27627 |
| 2016 | BMI | Female | 0-2 years | 16607 | 15672 |
| 2017 | Weight | Female | 0-2 years | 69412 | 59952 |
| 2017 | Height/length | Female | 0-2 years | 60996 | 57551 |
| 2017 | BMI | Female | 0-2 years | 16035 | 15201 |
| 2017 | HC | Female | 0-2 years | 59291 | 56581 |
| 2017 | Weight | Male | > 2 years | 60786 | 59988 |
| 2017 | Height/length | Male | > 2 years | 59639 | 59260 |
| 2017 | HC | Male | > 2 years | 28492 | 28376 |
| 2017 | BMI | Male | > 2 years | 52706 | 52408 |
| 2017 | Weight | Male | 0-2 years | 72839 | 63331 |
| 2017 | Height/length | Male | 0-2 years | 64428 | 61009 |
| 2017 | HC | Male | 0-2 years | 62670 | 59950 |
| 2017 | BMI | Male | 0-2 years | 16528 | 15704 |
| 2017 | Weight | Female | > 2 years | 57800 | 57100 |
| 2017 | Height/length | Female | > 2 years | 56689 | 56353 |
| 2017 | BMI | Female | > 2 years | 50142 | 49832 |
| 2017 | HC | Female | > 2 years | 26897 | 26779 |
| 2018 | Weight | Male | > 2 years | 58628 | 57824 |
| 2018 | Height/length | Male | > 2 years | 57324 | 56976 |
| 2018 | BMI | Male | > 2 years | 50826 | 50519 |
| 2018 | Weight | Male | 0-2 years | 68776 | 60321 |
| 2018 | Height/length | Male | 0-2 years | 61260 | 58295 |
| 2018 | HC | Male | 0-2 years | 59723 | 57340 |
| 2018 | Weight | Female | > 2 years | 55343 | 54630 |
| 2018 | Height/length | Female | > 2 years | 54115 | 53814 |
| 2018 | BMI | Female | > 2 years | 48099 | 47870 |
| 2018 | Weight | Female | 0-2 years | 66039 | 57434 |
| 2018 | Height/length | Female | 0-2 years | 58224 | 55383 |
| 2018 | BMI | Female | 0-2 years | 14392 | 13736 |
| 2018 | HC | Female | 0-2 years | 56647 | 54460 |
| 2018 | HC | Male | > 2 years | 27352 | 27223 |
| 2018 | HC | Female | > 2 years | 25717 | 25608 |
| 2018 | BMI | Male | 0-2 years | 15185 | 14552 |
| 2019 | Weight | Female | > 2 years | 53255 | 52449 |
| 2019 | Height/length | Female | > 2 years | 51872 | 51523 |
| 2019 | BMI | Female | > 2 years | 45868 | 45604 |
| 2019 | HC | Female | > 2 years | 24353 | 24241 |
| 2019 | Weight | Female | 0-2 years | 63839 | 55138 |
| 2019 | Height/length | Female | 0-2 years | 55842 | 53127 |
| 2019 | BMI | Female | 0-2 years | 12981 | 12381 |
| 2019 | HC | Female | 0-2 years | 54329 | 52235 |
| 2019 | Weight | Male | > 2 years | 56362 | 55601 |
| 2019 | Height/length | Male | > 2 years | 55013 | 54687 |
| 2019 | BMI | Male | > 2 years | 48788 | 48546 |
| 2019 | HC | Male | > 2 years | 25621 | 25508 |
| 2019 | Weight | Male | 0-2 years | 66960 | 58483 |
| 2019 | Height/length | Male | 0-2 years | 59155 | 56421 |
| 2019 | HC | Male | 0-2 years | 57654 | 55445 |
| 2019 | BMI | Male | 0-2 years | 13763 | 13171 |

# Supplementary Table 4. Baseline characteristics of total included sample (2013-2019) versus initial sample

|  | Inital sample | Included sample |
| --- | --- | --- |
| N | 1,193,563 | 560,476 |
| MEDEA |  |  |
| Rural | 314,185 (26.32%) | 153,732 (27.43%) |
| Urban least deprived | 169,539 (14.20%) | 72,417 (12.92%) |
| Urban 2Q | 177,629 (14.88%) | 81,751 (14.59%) |
| Urban 3Q | 265,727 (22.26%) | 126,693 (22.60%) |
| Urban most deprived | 266,483 (22.33%) | 125,883 (22.46%) |
| Nationality |  |  |
| Others | 130,532 (10.94%) | 48,220 (8.60%) |
| Spanish | 1,063,031 (89.06%) | 512,256 (91.40%) |
| Sex |  |  |
| Female | 592,040 (49.60%) | 271,919 (48.52%) |
| Male­ | 601,523 (50.40%) | 288,557 (51.48%) |

# Supplementary Figure 4. Male: Length / height 0-15 years


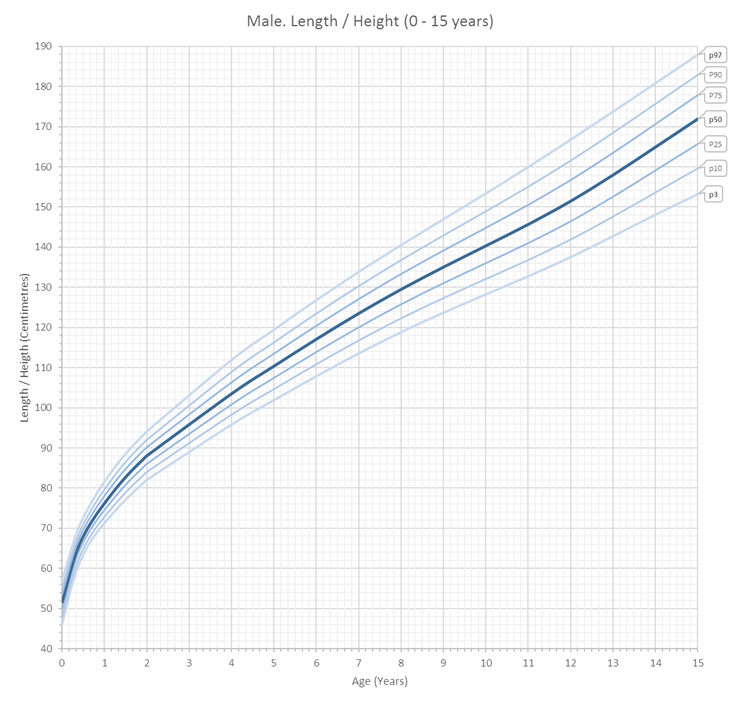


# Supplementary Figure 5. Male: Length 0-24 months


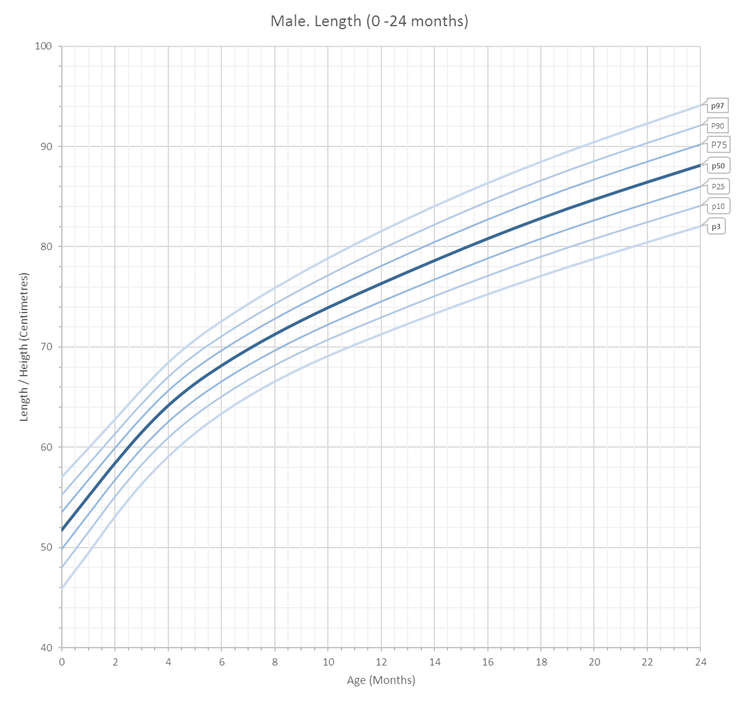


# Supplementary Figure 6. Male: Weight 0-15 years


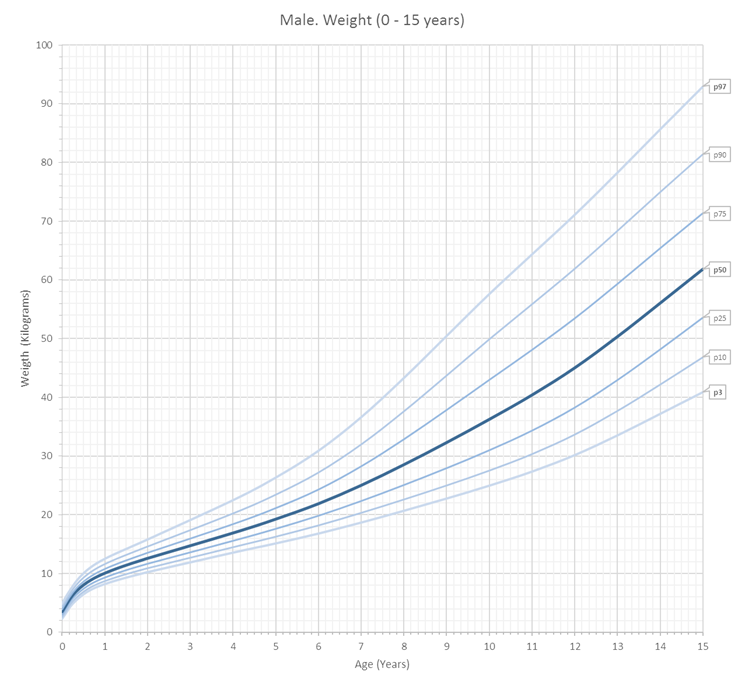


# Supplementary Figure 7. Male: Weight 0-24 months 
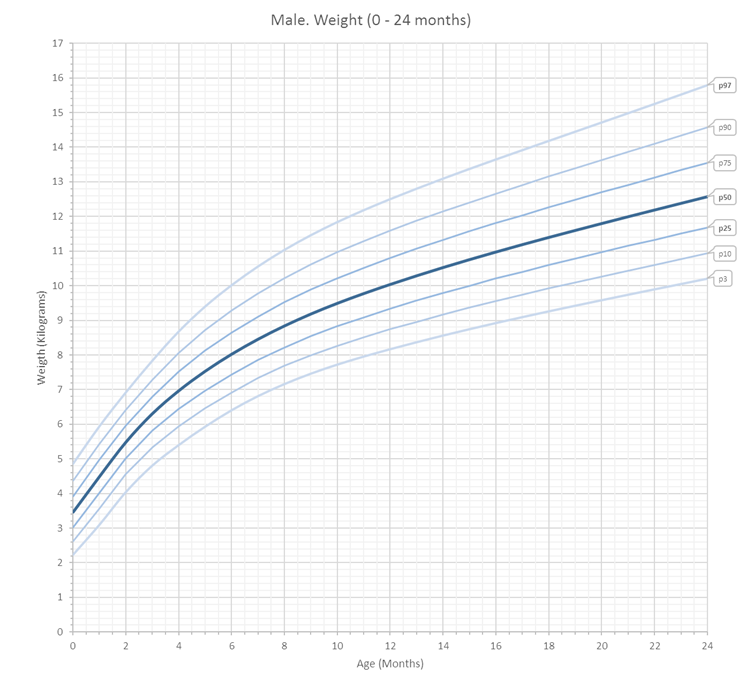


# Supplementary Figure 8. Male: Body mass index 4-15 years


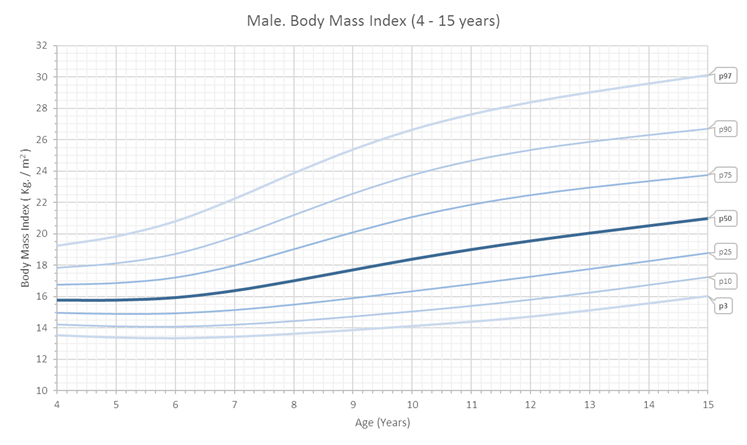


# Supplementary Figure 9. Male: Head circumference 0-24 months


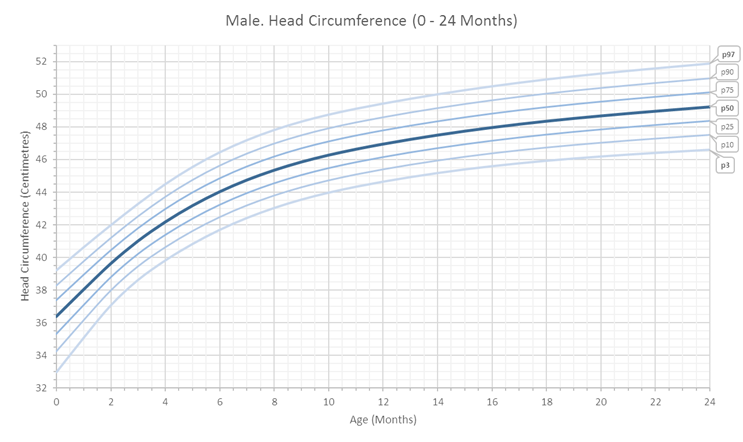


Supplementary Figure 10. Female: Length / height 0-15 years
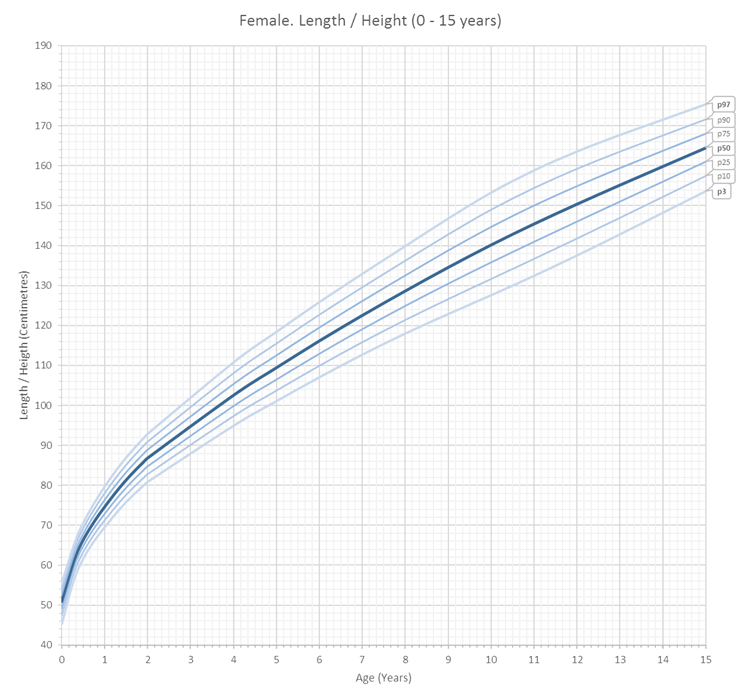


Supplementary Figure 11. Female: Length 0-24 months 
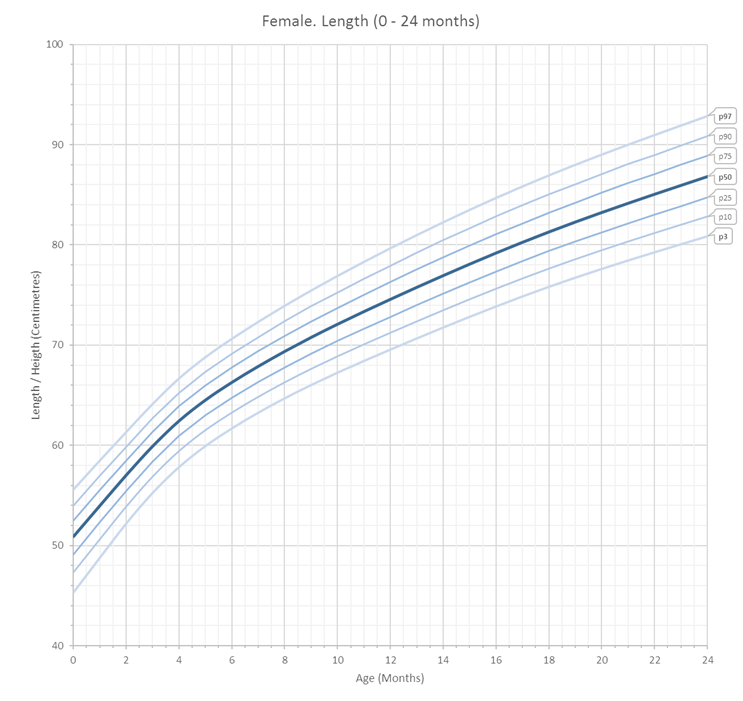


# Supplementary Figure 12. Female: Weight 0-15 years 
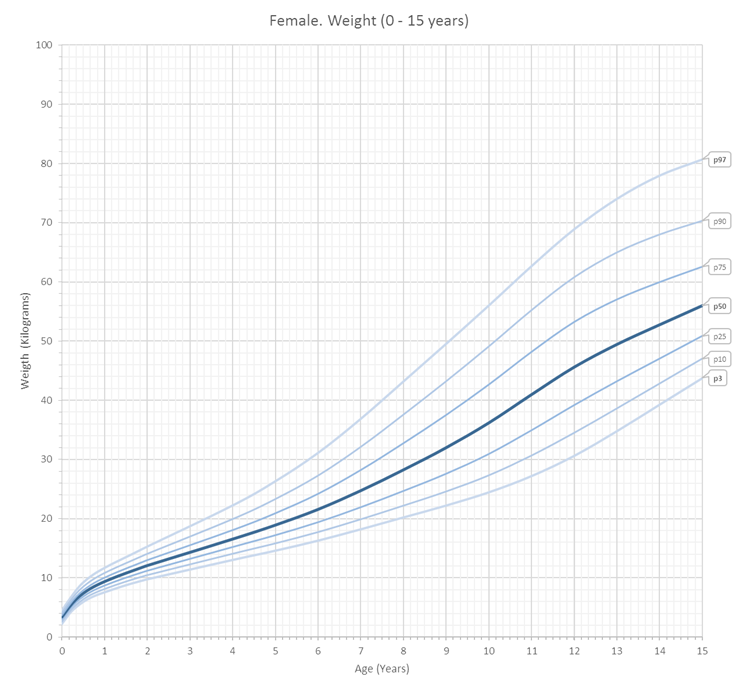


Supplementary Figure 13. Female: Weight 0-24 months 
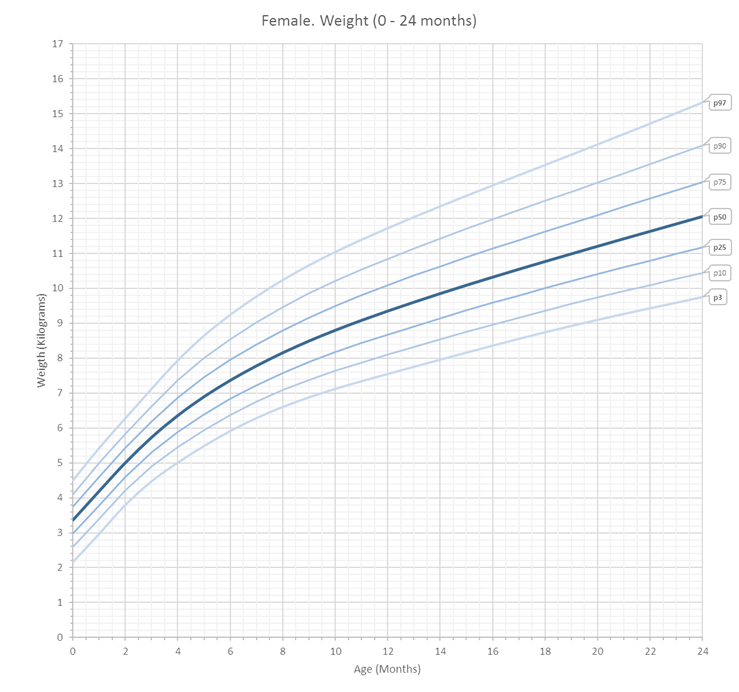


Supplementary Figure 14. Female: Body mass index 4-15 years 
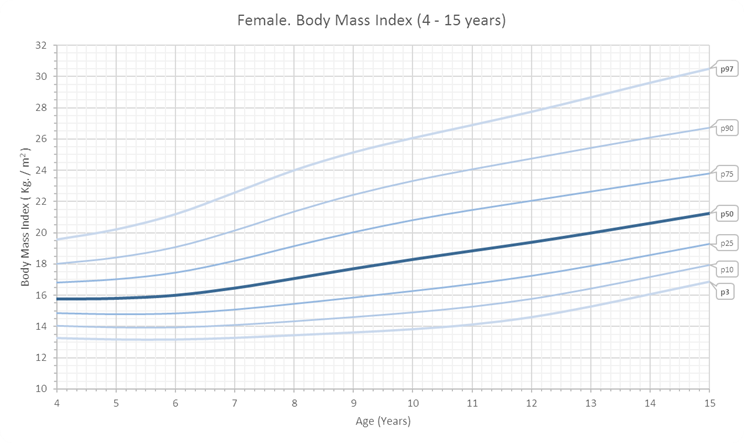


Supplementary Figure 15. Female: Head circumference 0-24 months 
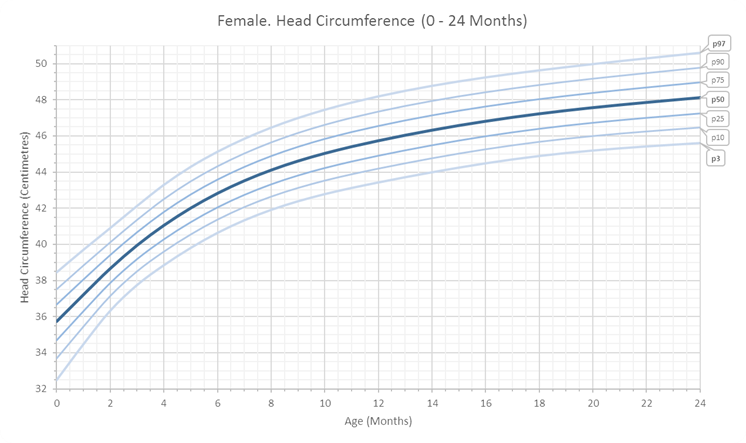


# Supplementary Figure 16. Worm plot: Weight females 0-24 months


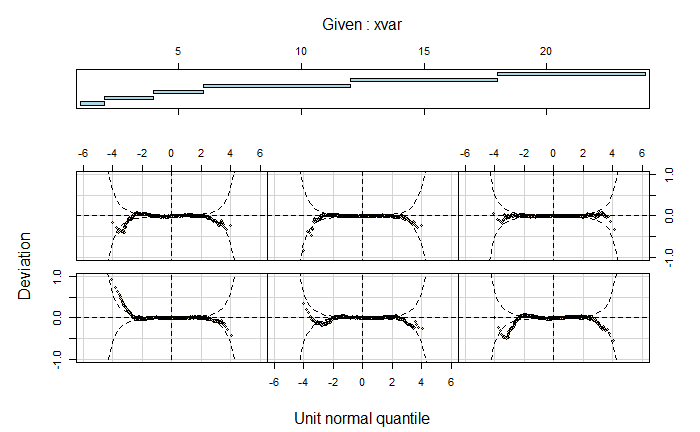


# Supplementary Figure 17. Worm plot: Length females 0-24 months


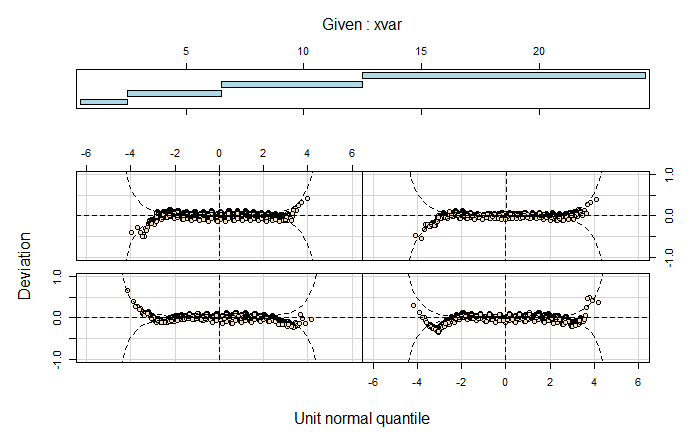


# Supplementary Figure 18. Worm plot: Head circumference females 0-24 months


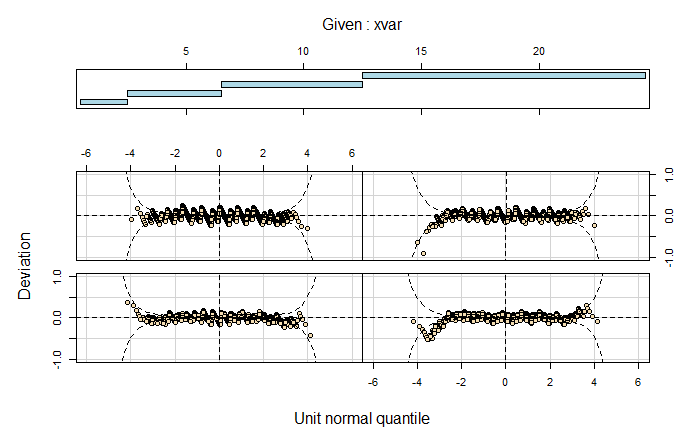


# Supplementary Figure 19. Worm plot: Weight males 0-24 months


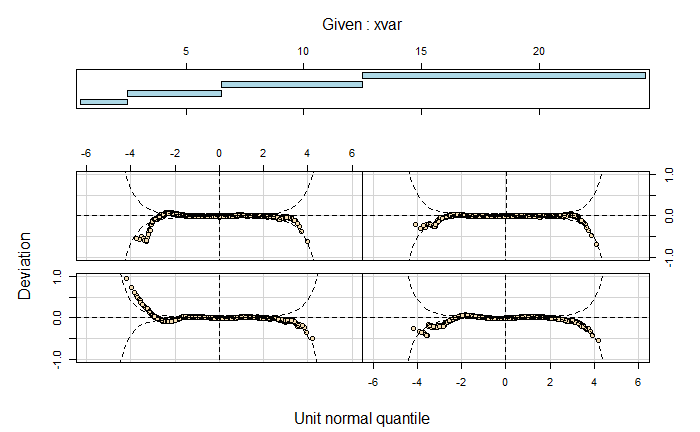


# Supplementary Figure 20. Worm plot: Length males 0-24 months


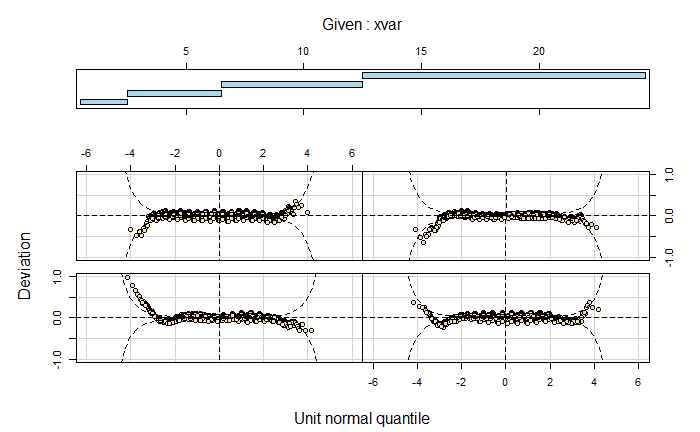


# Supplementary Figure 21. Worm plot: Head circumference males 0-24 months


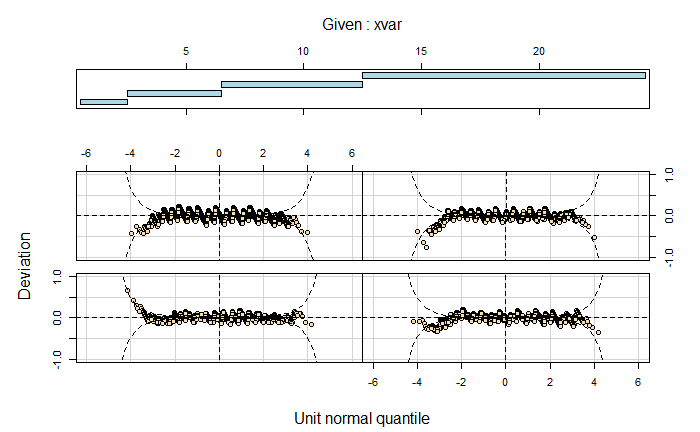


# Supplementary Figure 22. Worm plot: Weight females 4-14 years


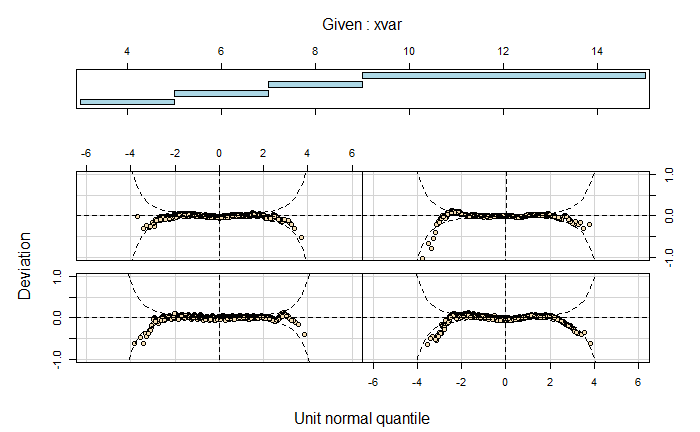


# Supplementary Figure 23. Worm plot: Height females 4-14 years


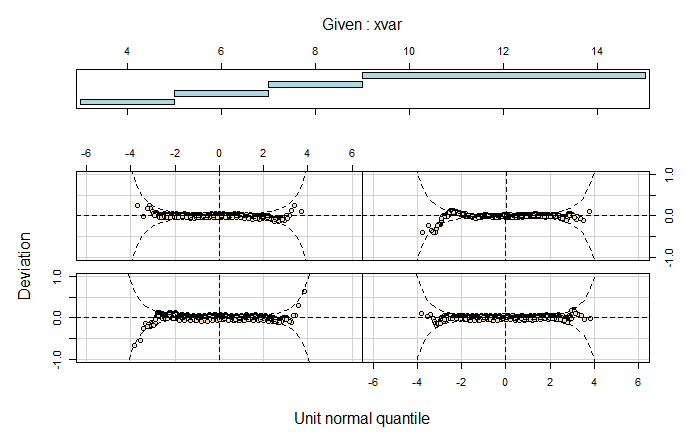


# Supplementary Figure 24. Worm plot: BMI females 4-14 years

#
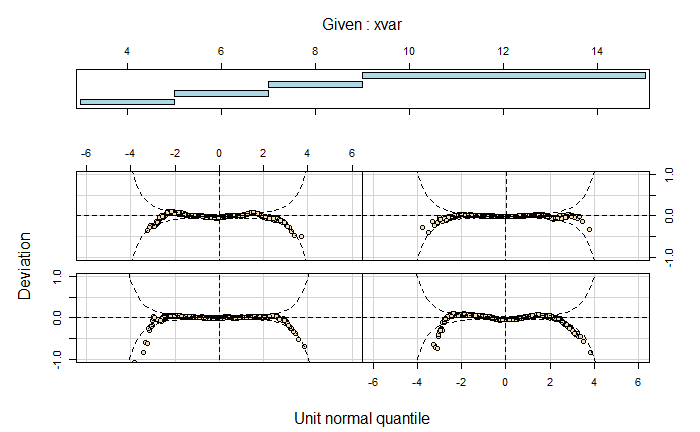
Supplementary Figure 25. Worm plot: Weight males 4-14 years


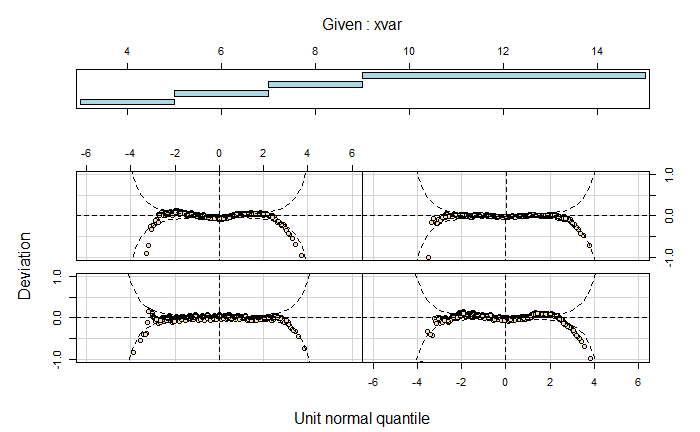


# Supplementary Figure 26. Worm plot: Height males 4-14 years


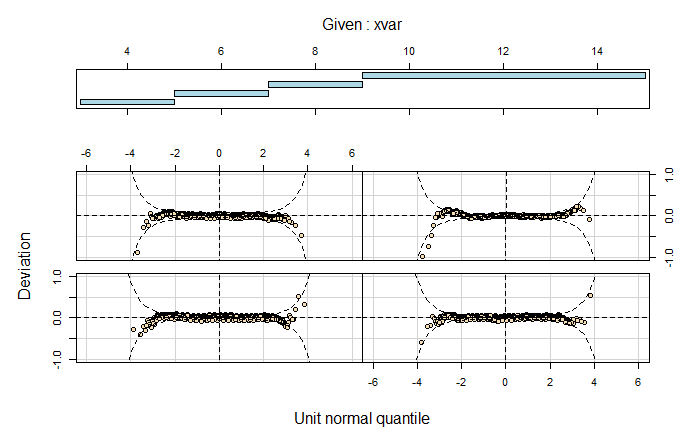


# **Supplementary Figure 27. Worm plot: BMI males 4-14 years**


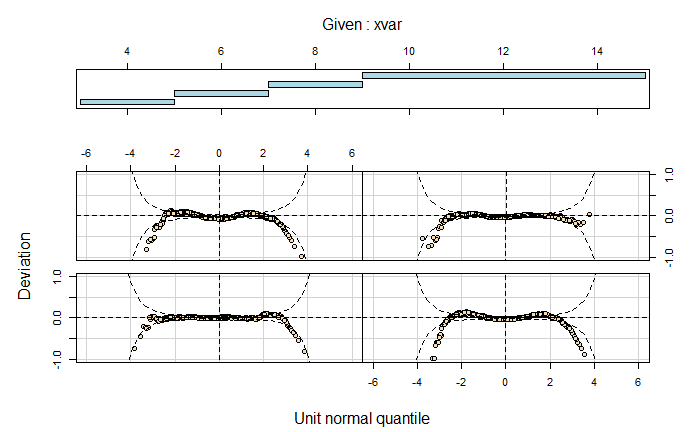

Supplement: Supplementary file 1 — Supplementary Material 1. [file 12889_2026_27263_MOESM1_ESM.docx]
